# Supplementary material for: Sorption Constant of Bisphenol A and Octylphenol Onto Size-Fractioned Dissolved Organic Matter Using a Fluorescence Method
Source: Int J Environ Res Public Health. 2021 Jan 27;18(3):1102. doi: 10.3390/ijerph18031102 (PMC7908396; doi:10.3390/ijerph18031102)
Supplement: Supplementary file 1 [file ijerph-18-01102-s001.pdf]

International Journal of
Environmental Research and Public Health

MDPI

Supplementary Materials

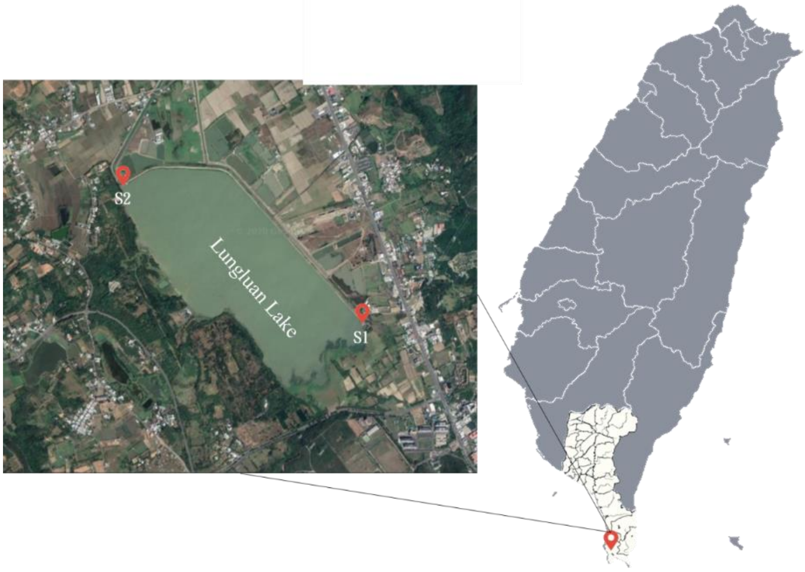

Figure S1. Sampling map.

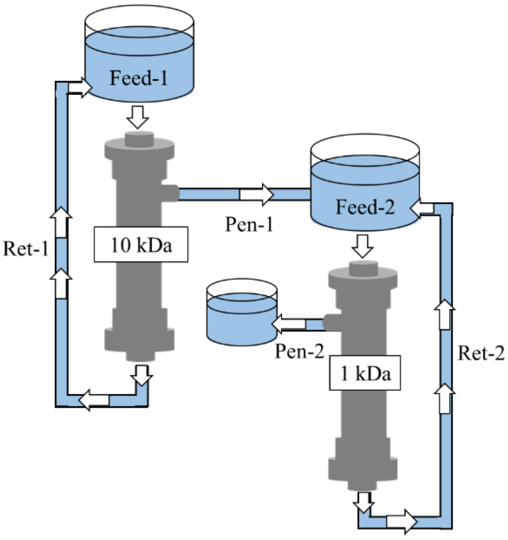

Size	Feed	Retention	Penetration
100 kDa	3000 mL (Feed-1)	300 mL (Ret-1, HDOM)	2700 mL (Pen-1, Feed-2)
1 kDa	2700 mL (Feed-2)	270 mL (Ret-2, MDOM)	2430 mL (Pen-2, LDOM)

Figure S2. Separation process and volumes of sized DOM.

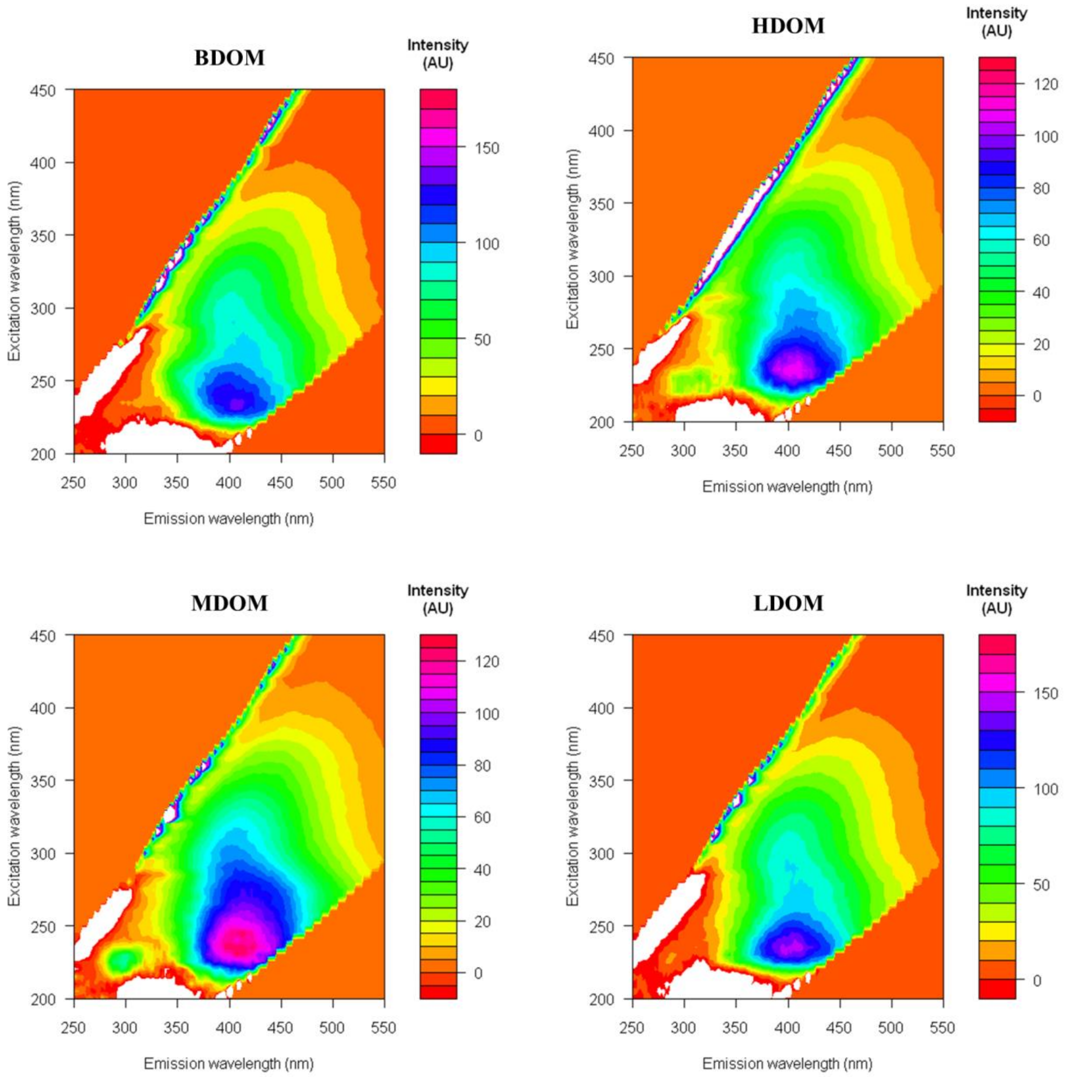

Figure S3. EEM plots of 4 sized DOM without OP and BPA at DOC 1 mg/L.

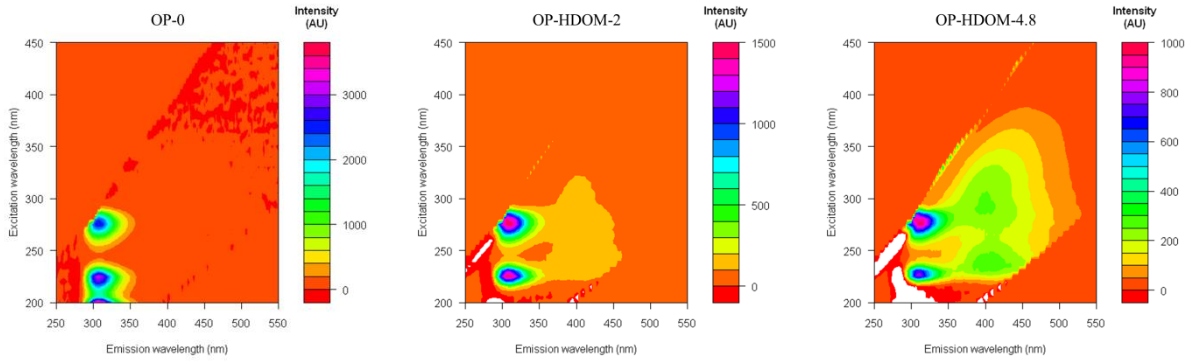

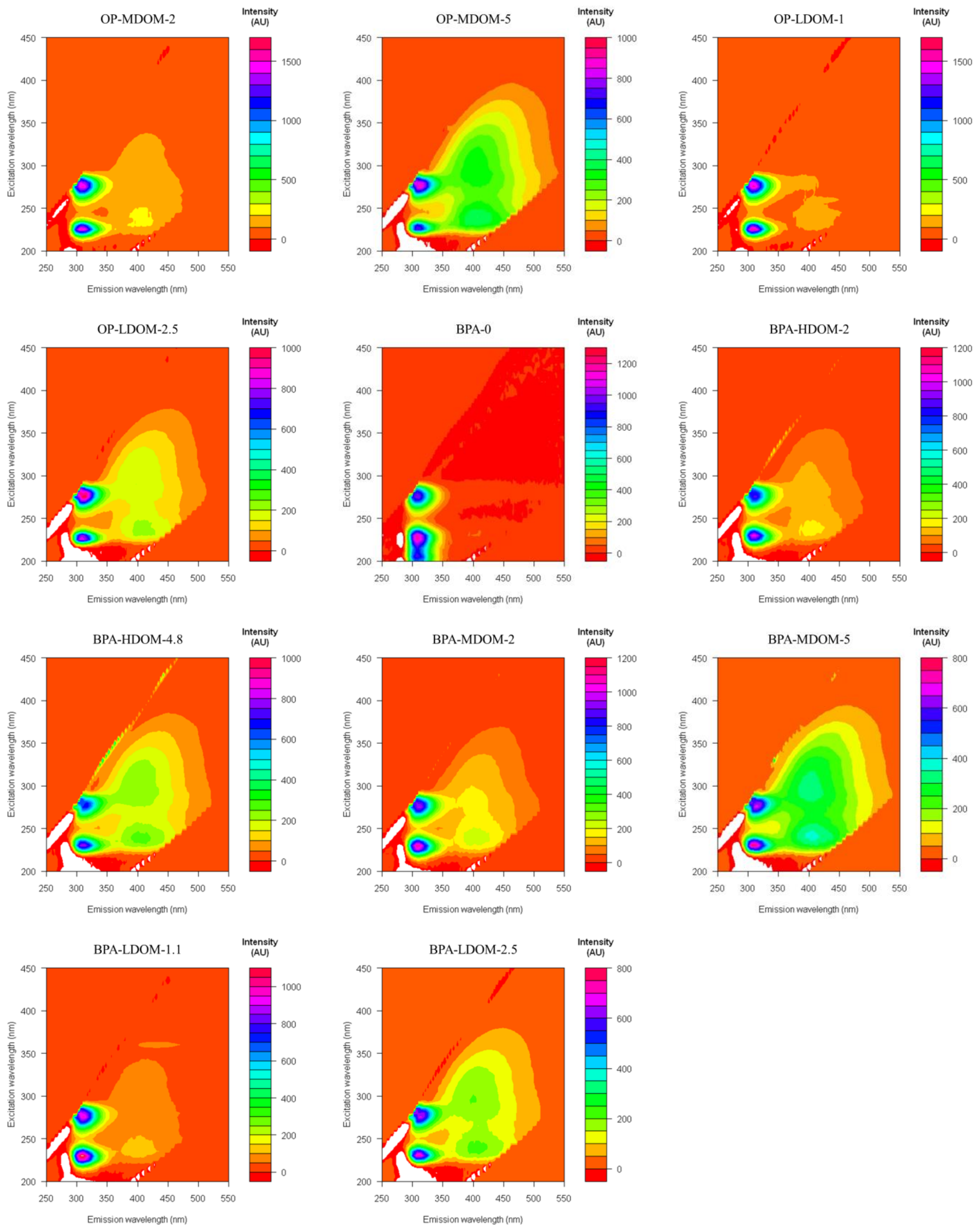

Figure S4. EEM plots of OP/BPA interaction with size-fractioned DOM. In the top of EEM, OP-0 and BPA-0 were the EEM plots of standard OP/BPA solution (1 mg/L). The size-fractions DOM were represented with HDOM, MDOM, and LDOM. The numbers were the DOC concentrations.
